# Expression of protocadherin gamma in skeletal muscle tissue is associated with age and muscle weakness

**DOI:** 10.1002/jcsm.12099

**Published:** 2016-02-02

**Authors:** Roland W. J. Hangelbroek, Parastoo Fazelzadeh, Michael Tieland, Mark V. Boekschoten, Guido J. E. J. Hooiveld, John P. M. van Duynhoven, James A. Timmons, Lex B. Verdijk, Lisette C. P. G. M. de Groot, Luc J. C. van Loon, Michael Müller

**Affiliations:** ^1^ Top Institute Food and NutritionWageningenthe Netherlands; ^2^ Division of Human NutritionWageningen UniversityWageningenthe Netherlands; ^3^ Laboratory of BiophysicsWageningen UniversityWageningenthe Netherlands; ^4^ Netherlands Metabolomics CentreLeidenthe Netherlands; ^5^ King's College LondonLondonUK; ^6^ XRgenomics Ltd.LondonUK; ^7^ Department of Human Movement Sciences, NUTRIM School of Nutrition and Translational Research in MetabolismMaastricht UniversityMaastrichtthe Netherlands; ^8^ Norwich Medical SchoolUniversity of East AngliaNorwichUK

**Keywords:** Ageing, Frailty, Skeletal muscle, Transcriptomics

## Abstract

**Background:**

The skeletal muscle system plays an important role in the independence of older adults. In this study we examine differences in the skeletal muscle transcriptome between healthy young and older subjects and (pre‐)frail older adults. Additionally, we examine the effect of resistance‐type exercise training on the muscle transcriptome in healthy older subjects and (pre‐)frail older adults.

**Methods:**

Baseline transcriptome profiles were measured in muscle biopsies collected from 53 young, 73 healthy older subjects, and 61 frail older subjects. Follow‐up samples from these frail older subjects (31 samples) and healthy older subjects (41 samples) were collected after 6 months of progressive resistance‐type exercise training. Frail older subjects trained twice per week and the healthy older subjects trained three times per week.

**Results:**

At baseline genes related to mitochondrial function and energy metabolism were differentially expressed between older and young subjects, as well as between healthy and frail older subjects. Three hundred seven genes were differentially expressed after training in both groups. Training affected expression levels of genes related to extracellular matrix, glucose metabolism ,and vascularization. Expression of genes that were modulated by exercise training was indicative of muscle strength at baseline. Genes that strongly correlated with strength belonged to the protocadherin gamma gene cluster (*r* = −0.73).

**Conclusions:**

Our data suggest significant remaining plasticity of ageing skeletal muscle to adapt to resistance‐type exercise training. Some age‐related changes in skeletal muscle gene expression appear to be partially reversed by prolonged resistance‐type exercise training. The protocadherin gamma gene cluster may be related to muscle denervation and re‐innervation in ageing muscle.

## Introduction

The number of people aged 65 and above has increased rapidly over the past few decades and is likely to increase progressively.^1^ Because senescence is associated with a wide range of afflictions, including physical disability, cancer, heart disease, and diabetes, the demand for care for older people will further increase. The loss of skeletal muscle mass and function with ageing leads to frailty and results in the loss of independence of older adults.

Frailty and related sarcopenia are very complex, and many factors contribute to their aetiology. This includes physical inactivity, malnutrition, hormonal changes, and changes within the muscle.^2^, ^3^, ^4^ Mitochondrial function decreases with age,^5^ fast‐twitch muscle fibres demonstrate a smaller cross‐sectional area,^6^ protein synthesis capacity is reduced,^7^ anabolic signals are less effective,^4^, ^8^ and there are fewer satellite cells to regulate adaptive responses to stimuli.^9^ In older adults muscle cells can also undergo continuous cycles of denervation and reinnervation, which can lead to both weakness and loss of muscle mass.^10^, ^11^

One of the most effective strategies to improve muscle mass and strength in adults is physical exercise.^9^, ^12^ Resistance‐type exercise is particularly suitable to curtail muscle loss and muscle weakness in older people. In accordance, quality of life is improved after participating in resistance‐type exercise training.^13^ Some even claim that resistance‐type exercise training reverses ageing in skeletal muscle.^14^

To elucidate some of these complex processes that occur in skeletal muscle during ageing, we examined the effects of prolonged resistance‐type exercise training in frail and healthy older subjects on the skeletal muscle transcriptome. By comparing genome‐level gene expression in frail and pre‐frail older subjects, healthy older subjects, and young subjects we aim to better understand the molecular causes of frailty. Secondly, we aimed to determine the effect of resistance‐type exercise training on the skeletal muscle transcriptome in both frail and healthy older people.

## Methods

### Experimental design

We collected a total of 259 muscle biopsy samples from pre‐frail and frail older subjects (61 subjects, 92 samples), healthy older subjects (73 subjects, 114 samples), and young males (53 subjects, 53 samples). Some of these samples were follow‐up samples taken after 24 weeks of resistance‐type exercise training (31 samples from the frail older subjects, 41 samples from the healthy older subjects). Training for both groups was similar and consisted of progressive full‐body resistance‐type exercise training. However, the frail older group had training sessions twice per week, whereas the healthy older group trained three times per week. In addition, subjects took a protein or control drink for the duration of the study. The healthy older group received a 15 g portion of milk protein or control supplement at breakfast. The frail older group received a similar drink containing 15 g supplement drink (milk protein or control) at breakfast and lunch. More details can be found in the respective papers.^15^, ^16^ Table 1 shows the characteristics of our study population at baseline. Table 2 shows the effect of the training intervention on the older subjects that were included in this study and where follow‐up data are available.

**Table 1:** Subject characteristics of the baseline only subjects

	FE	HE	YO
*N (male / female)*	24 / 6	27 / 5	53 / 0
*Age (years)*	79.8 ± 8.9	74.1 ± 4.5	21.3 ± 2.4
*Height (m)*	1.71 ± 0.09	1.73 ± 0.08	1.84 ± 0.06
*Weight (kg)*	80 ± 12.4	75.9 ± 12.9	76.5 ± 10.3
*BMI (kg/m^2^)*	27.3 ± 4.2	25.2 ± 3.2	22.6 ± 3
*Body Fat (%)*	28.8 ± 7.2	23.4 ± 5.5	15.4 ± 4.6
*Lean Mass (kg)*	52.1 ± 6.3	55.5 ± 8.6	61.9 ± 6
*Leg Extension 1RM (kg)*	65 ± 20	68 ± 17	124 ± 20
*Leg Press 1RM (kg)*	127 ± 31	155 ± 41	203 ± 36

Mean ± SD. FE = frail older subjects, HE = healthy older subjects, YO = young male subjects.

**Table 2:** Subject characteristics of the subjects with before and after samples

	FE before training	FE after training	HE before training	HE after training
*N (male/female)*	11 / 20		26 / 15	
*Age (years)*	76.5 ± 7.0		69.9 ± 5.0	
*Height (m)*	1.66 ± 0.09		1.71 ± 0.09	
*Weight (kg)*	78.5 ± 13.6	79.6 ± 14.1*	76.7 ± 13.2	77.1 ± 13.2
*BMI (kg/m^2^)*	28.5 ± 4.1	29 ± 4.3 *	26.1 ± 2.8	26.2 ± 2.8
*Body Fat (%)*	36.7 ± 8.5	36.6 ± 8.8	25.9 ± 5.9	24.4 ± 5.9 *
*Lean Mass (kg)*	46.1 ± 10.0	46.9 ± 9.9 *	54.6 ± 11.1	56.0 ± 11.3 *
*Leg Extension 1RM (kg)*	59 ± 18	81 ± 24 *	81 ± 17	114 ± 23 *
*Leg Press 1RM (kg)*	130 ± 35	178 ± 49 *	179 ± 40	230 ± 50 *

Mean ± SD. FE = frail older subjects, HE = healthy older subjects, YO = young male subjects.

* A significant effect of resistance‐type exercise training (*P* < 0.05).

### Subjects

Biopsies from frail and pre‐frail older subjects were collected from participants of two studies performed by Tieland *et al.* 15, 17 For these studies frail and pre‐frail older subjects were selected based on the Fried criteria for frailty.^2^ These subjects will hereafter be referred to as frail older subjects. These characteristics are unintentional weight loss, weakness, self‐reported exhaustion, slow walking speed, and low physical activity. Subjects in the healthy older group were not considered frail by any of these criteria at the start of the intervention study.^16^ Several additional baseline samples from healthy older subjects were collected from several studies from our group.^18^, ^19^ These samples were taken before any intervention was undertaken and serve as additional reference samples. Baseline samples from young subjects were from healthy male subjects.^20^ These were also taken before any intervention took place and serve as reference samples. All studies were approved by the medical ethical committee of either Wageningen University or Maastricht University and comply with the Declaration of Helsinki.

### Muscle biopsy

Muscle samples were obtained with a 5 mm Bergstrom muscle biopsy needle from the *Musculus vastus lateralis*, after local anaesthesia of the skin and fascia. Samples were freed from any visible blood and non‐muscle tissue and immediately frozen in liquid nitrogen and then stored at −80°C. All samples were obtained in the morning, in an overnight fasted state, after at least 3 days of no heavy physical activity.

### Sample preparation and microarray analysis

Total RNA was isolated from the skeletal muscle tissue by using Trizol reagent (Invitrogen, Breda, Netherlands). Thereafter, RNA was purified using the Qiagen RNeasy Micro kit (Qiagen, Venlo, Netherlands), and RNA quality was checked using an Agilent 2100 bioanalyzer (Agilent Technologies, Amsterdam, Netherlands). Total RNA (100 ng) was labelled using an Ambion WT expression kit (Life Technologies, Bleiswijk, Netherlands) and hybridized to human whole genome Genechip Human Gene 1.1 ST arrays coding 19.732 genes, (Affymetrix, Santa Clara, CA). Sample labelling, hybridization to chips, and image scanning were performed according manufacturer's instructions.

### Data analysis

**Figure 1:**
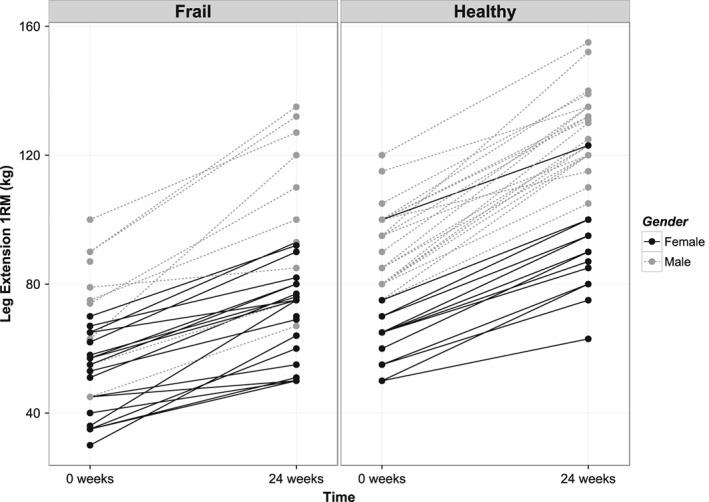
A and B—Change of leg extension 1RM after prolonged exercise training for each individual. Left are frail older subjects, right are healthy older subjects.

Microarray signals were normalized using robust multichip average. Data were filtered using Universal exPression Codes filtering with a 50% cut‐off, corresponding to a 50% likelihood that a gene is expressed.^21^ Significant differences of individual genes were tested using the limma R library.^22^ Baseline differences were tested between the three groups (frail older, healthy older, or young). Our model included gender, supplementation, and group. For the effect of exercise we included subject, gender, time, and supplementation in the model. The training effect for frail older and healthy older subjects was analysed separately because of differences in training frequency. *P*‐values were adjusted using false discovery rate.^23^ A *q*‐value below 0.05 was considered significant. Pathway analyses were performed using Ingenuity Pathway Analysis (IPA, QIAGEN Redwood City, www.qiagen.com/ingenuity) on the filtered dataset with the Universal exPression Codes filtering filtered genes used as the background. A sparse partial least squares (sPLS) model for leg extension 1RM (*Figure* 1) was made using the caret R library.^24^ The dataset was split into a training set (75%) and a testing set (25%) before fitting the model using cross validation. This model was validated using 10 times repeated tenfold cross validation. Final number of components for the sPLS model selected by grid search was 3. Principal component analysis was performed using the FactoMineR R library.^25^ Plots were made using the R libraries ggplot2 and gplots.^26^, ^27^

## Results

### Baseline differences in transcriptome

**Figure 2:**
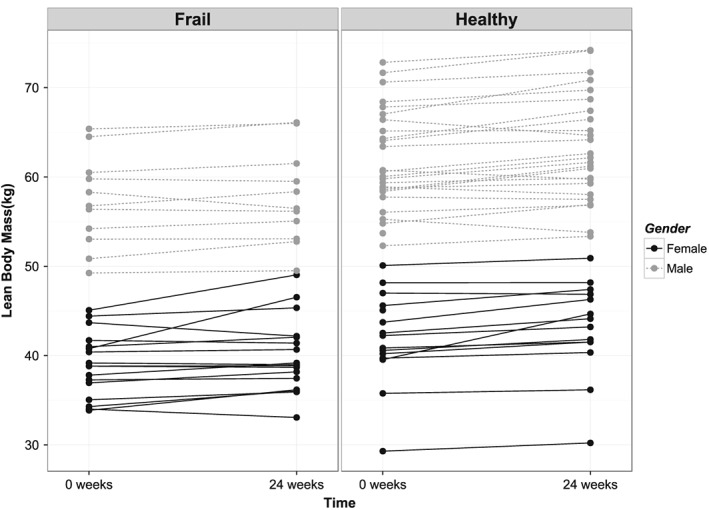
A and B—Change of lean body mass after prolonged exercise training for each individual. Left are frail older subjects, right are healthy older subjects.

Large differences in gene expression profiles between young and older adults (healthy and frail) were found, with the expression of 5228 genes significantly different between young subjects and both groups of older subjects. However, fold changes of the majority of these genes were relatively subtle, which suggested small but consistent differences between these groups. Only 825 genes out of these 5228 genes showed fold changes higher than 1.2. Venn‐diagrams can be found in the Supporting Information, *Figures* S1A and S1B. The top 20 genes that were significantly different at baseline between the three groups are presented in Table 3. Top canonical pathways reported by IPA include oxidative phosphorylation, TCA cycle and glucose metabolism (Supporting Information, *Figure* S2) (*Figure* 2).

**Table 3:** Top 20 genes significantly different between young and older subjects

Gene	FC FE vs. HE	FC FE vs. YO	FC HE vs. YO	q‐value FE vs. HE	q‐value FE vs. YO	q‐value HE vs. YO
*IGFN1*	−1.12	−6.43	−5.71	0.64	0.00	0.00
*UNC13C*	1.07	6.20	5.78	0.60	0.00	0.00
*MYLK4*	−1.35	−5.13	−3.81	0.07	0.00	0.00
*C12orf75*	1.45	4.54	3.13	0.01	0.00	0.00
*SLC38A1*	−1.11	−3.50	−3.14	0.55	0.00	0.00
*HCN1*	1.16	3.36	2.90	0.30	0.00	0.00
*MYH8*	1.16	3.28	2.83	0.50	0.00	0.00
*CFAP61*	1.36	3.39	2.49	0.00	0.00	0.00
*NR4A3*	−1.86	−3.70	−2.00	0.02	0.00	0.00
*FAM83B*	1.11	2.91	2.63	0.39	0.00	0.00
*DAAM2*	−1.06	−2.51	−2.36	0.42	0.00	0.00
*NNMT*	1.33	2.71	2.04	0.03	0.00	0.00
*ZNF382*	1.21	−2.09	−2.54	0.05	0.00	0.00
*TPPP3*	1.33	2.62	1.97	0.01	0.00	0.00
*COL28A1*	1.07	2.36	2.20	0.42	0.00	0.00
*METTL21EP*	1.05	−2.19	−2.29	0.78	0.00	0.00
*HIST1H3E*	1.11	−2.08	−2.30	0.30	0.00	0.00
*SNORD115‐32*	1.02	2.17	2.14	0.91	0.00	0.00
*SERPINA5*	1.04	−2.11	−2.19	0.89	0.00	0.00
*METTL21C*	1.98	2.84	1.44	0.00	0.00	0.03

FE = frail older subjects, HE = healthy older subjects, YO = young men.

Table 4 shows the top 20 genes that were different between healthy and frail older subjects at baseline. Top genes included METTL21C, FRZB, and non‐coding RNAs. Pathways that were significantly different between the frail and healthy older subjects were related to glucose metabolism and RNA processing (Supporting Information, *Figure* S2). In general, expression of genes related to glucose metabolism were lower in both frail older and healthy older subjects compared with young, with frail older subjects showing the lowest expression of the groups. Principal component analysis summarizes this observation, where the healthy older subjects seemed to be between the frail older subjects and the young subjects on the first two components (Supporting Information, *Figures* S3 and S4). While pathways related to mitochondrial function were some of the most significantly affected pathways, the fold changes of the individual mitochondrial genes were relatively small but consistent. Fold changes for these mitochondrial genes were in the range of 1.1 and 1.2.

**Table 4:** Top 20 genes significantly different between the frail and healthy older adults at baseline

Gene	FC FE vs. HE	*q*‐value FE vs. HE
*METTL21C*	1.98	0.00
*NR4A3*	−1.86	0.02
*VTRNA1‐1*	−1.71	0.00
*MIR206*	1.58	0.00
*SNORA38B*	−1.58	0.00
*S100A8*	1.51	0.04
*FRZB*	−1.48	0.03
*HES1*	−1.47	0.00
*P2RY13*	1.46	0.00
*MIR133B*	1.45	0.01
*C12orf75*	1.45	0.01
*SNORA60*	−1.44	0.00
*SNORD60*	−1.43	0.00
*LYZ*	1.43	0.03
*SNORD80*	−1.42	0.00
*SNORD82*	−1.42	0.00
*SNORD29*	−1.41	0.00
*EVI2B*	1.40	0.01
*UPK3A*	−1.40	0.00
*ID1*	−1.40	0.00

FE = frail older subjects, HE = healthy older subjects.

### Effect of prolonged resistance‐type exercise training

Prolonged resistance‐type exercise training showed a significant effect on the gene expression profiles in both frail and healthy older people (431 and 1395 significantly changed genes, respectively). Three hundred seven genes were changed in both groups after resistance‐type exercise training. Changes in expression of all these overlapping genes were in the same direction. A table with the top 20 genes changed by training is presented in Table 5. Training resulted in the differential expression levels of many genes that are related to the connective tissue and the extracellular matrix, including collagen genes and laminin genes, suggesting significant tissue remodelling because of the training. Upstream analysis using IPA showed that TGF‐β signalling‐related genes were significantly activated in both groups, primarily because of the increased expression of collagen and laminin genes (Supporting Information, *Figure* S5). Other significant genes include myofibrillar proteins such as myosin heavy chain isoforms and troponin isoforms.

**Table 5:** Top 20 genes significantly different in both frail and healthy older subjects after training. FE = frail older subjects, HE = healthy older subjects

Gene	FC FE training	FC HE training	q‐value FE training	q‐value HE training
*FRZB*	1.97	1.55	0.00	0.00
*IGFN1*	1.58	1.80	0.04	0.00
*MYLK4*	1.45	1.69	0.01	0.00
*COL3A1*	1.45	1.68	0.01	0.00
*ANKRD2*	−1.44	−1.61	0.01	0.00
*THBS4*	1.34	1.66	0.05	0.00
*PFKFB3*	1.61	1.38	0.01	0.01
*COL4A1*	1.35	1.46	0.00	0.00
*CAPN6*	1.37	1.45	0.03	0.00
*COL1A2*	1.35	1.45	0.03	0.00
*EDNRB*	1.24	1.56	0.01	0.00
*GCNT2*	−1.51	−1.28	0.00	0.00
*CFAP61*	−1.24	−1.54	0.03	0.00
*C12orf75*	−1.42	−1.33	0.01	0.00
*CCDC80*	1.34	1.40	0.03	0.00
*OLFML2B*	1.38	1.34	0.00	0.00
*SPARC*	1.28	1.44	0.00	0.00
*COL4A2*	1.32	1.37	0.00	0.00
*LGI1*	−1.31	−1.33	0.02	0.00
*ACOT11*	−1.30	−1.34	0.04	0.00

**Figure 3:**
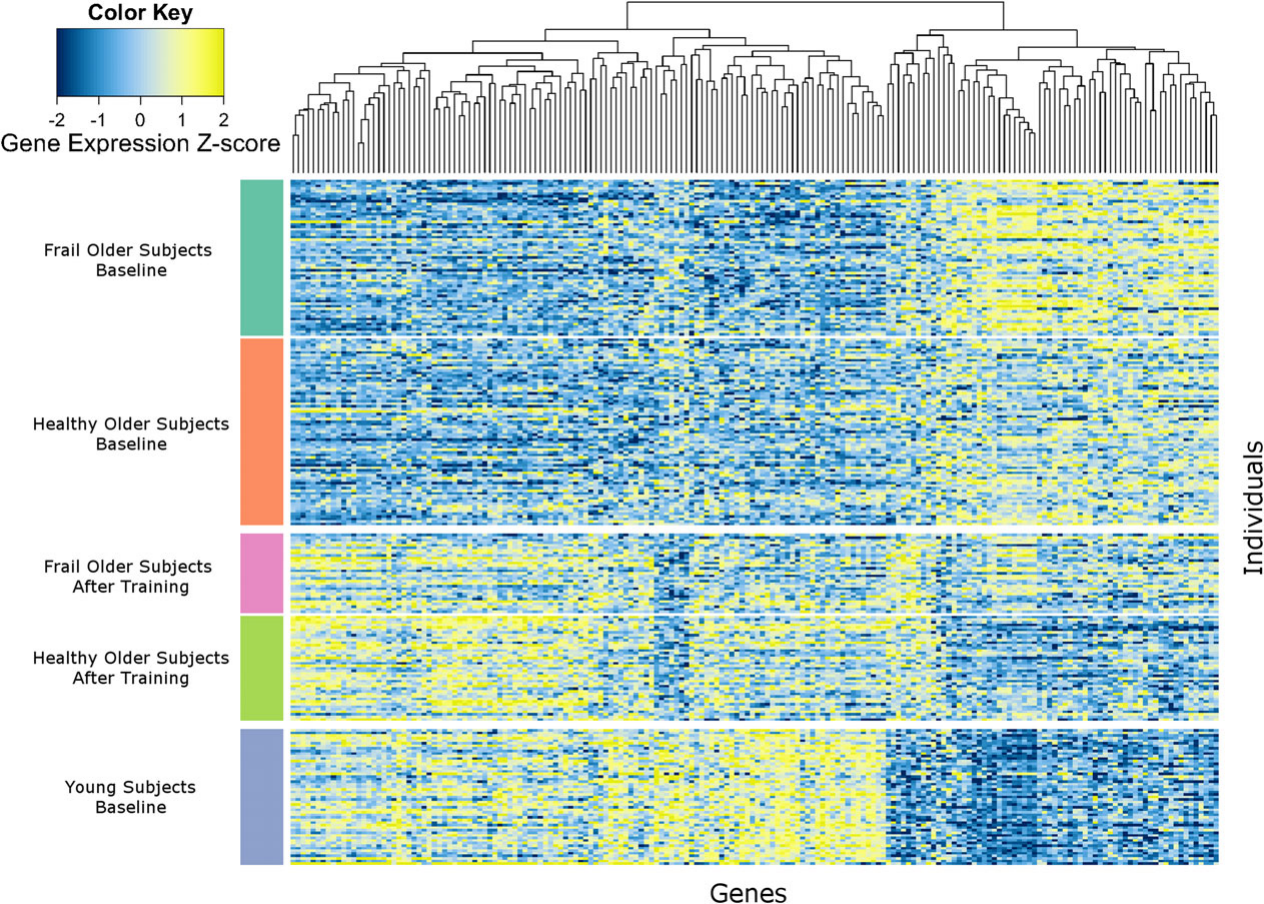
Heatmap of 184 genes that are significantly different between young and older subjects and are significantly changed by prolonged resistance‐type exercise training in both groups.

Genes related to glucose metabolism shifted away from the expression levels of the older subjects at baseline towards the levels of the younger phenotype. This trend is reflected in many other genes, where the majority of genes significantly changed by exercise training shifted towards ‘younger’ expression levels (325 genes out of 431 in the frail older subjects, 1106 out of 1395 in the healthy older subjects). *Figure* 3 shows a heat map of 184 genes that were significantly changed by training in both groups and were significant when comparing young with either frail or healthy older subjects. Most of these genes shifted towards younger levels.

**Figure 4:**
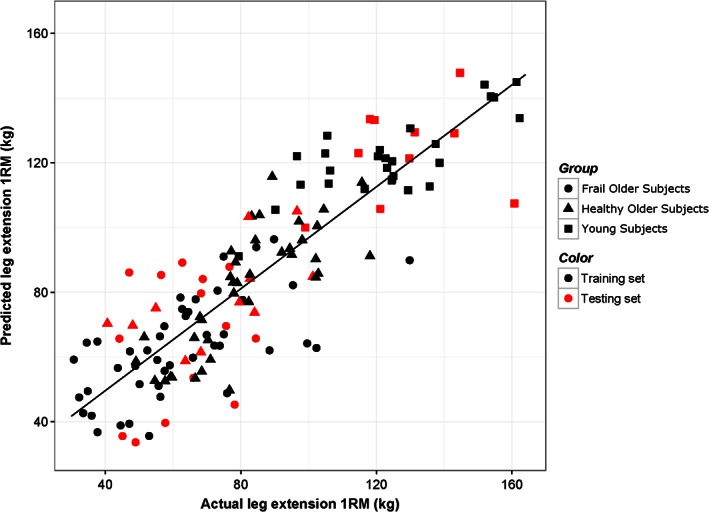
Scatter plot of predicted leg extension 1RM of the baseline samples using sPLS and the actual measured 1RM. Red dots indicate samples that were part of the testing data set (25% of the total data set).

To further analyse the relationship between the 307 genes that were robustly changed after training in both groups (*q*‐value < 0.05) we performed sPLS regression to calculate leg extension 1RM based on gene expression in the baseline samples. The aim was to evaluate whether differences in expression of the genes that were changed by training represent the overall strength of the muscle at baseline. The samples obtained after training were excluded for this analysis. A plot of the predicted leg extension 1RM strength against the measured leg extension 1RM strength is presented in *Figure* 4. Gene importance for the final model is presented in Table 6. Cross‐validation mean *R* ^2^ of the model was 0.73; the mean RMSE was 17.7. The RMSE for the withheld testing set was 19.1. The top genes contributing to the model include genes from the protocadherin gamma gene cluster, CTNNBIP1, CFAP61 (C20orf26), C12orf75, and USP54. We calculated the eigengene for all protocadherin gamma genes and correlated this eigengene with leg extension 1RM. The protocadherin gamma eigengene showed a strong direct negative correlation (Pearson *r* = −0.73) with 1RM leg extension strength. A plot of this negative correlation is presented in the Supporting Information, *Figure* S7.

**Table 6:** Variable importance and coefficients of the top 20 variables for the sPLS model

Gene	Variable importance	Coefficient
*CFAP61*	100	−1.94
*PCDHGA10*	99.3	−0.82
*PCDHGB5*	97.6	−1.18
*PCDHGB1*	95.1	−1.02
*CTNNBIP1*	91.9	1.12
*USP54*	90.6	−0.86
*PCDHGA8*	83.5	−0.91
*PCDHGB7*	82.4	−0.80
*MYOZ2*	81.3	−0.61
*PCDHGA11*	78.5	−0.64
*C12orf75*	78.2	−0.82
*PCDHGA7*	77.8	−0.86
*PCDHGA2*	76.4	−0.91
*HEXIM2*	75.9	0.24
*GRSF1*	75.2	0.39
*GCNT2*	75.1	−1.06
*FBP2*	72.3	0.26
*PLEKHO1*	68.5	0.24
*CRY2*	68.1	−1.60
*PABPC4*	67.5	0.85

## Discussion

**Figure 5:**
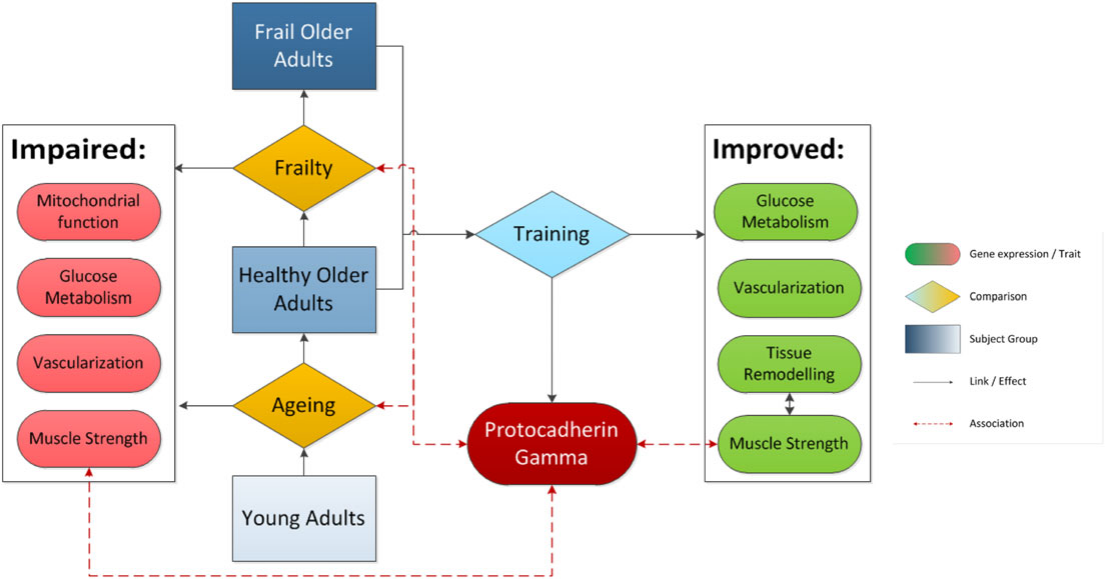
Scheme summarizing the major findings of this study.

In this study we compared the transcriptomes of skeletal muscle of healthy young, healthy older, and frail older subjects to better understand the skeletal muscle related part of the frail phenotype. A schematic overview of our findings is presented in *Figure* 5. To our knowledge this is the first study investigating the effect of age on the muscle transcriptome to include frail and pre‐frail older subjects. We observed clear and pronounced differences at baseline between young and older subjects. In our data frailty seems to present itself in the muscle transcriptome primarily as a more advanced stage of ageing (refer also to the Supporting Information, *Figures* S3 and S4). This may, at least partly, be because of the higher average age of the frail group. There is, however, still significant overlap in age because of the high variation in age in both groups (79.8 ± 8.9, 74.1 ± 4.5 mean age and standard deviation for frail and healthy older subjects, respectively; Tables 1 and 2).

### Baseline differences between young and older subjects

There were significant differences in genes related to mitochondrial function and oxidative phosphorylation (Supporting Information, *Figure* S2). It is well known that mitochondrial function is impaired in older adults,^5^ which can be a responsive feature to muscle inactivity^28^ and mitochondrial protein carbonylation.^29^ In this case the average expression of mitochondrial genes is lowest in the frail older subjects at baseline. These expression differences may also represent a lower abundance of mitochondria.^6^

There are two genes among the top differentially expressed genes between the three baseline groups that are as yet unknown, CFAP61 (C20orf26) and C12orf75. Both genes have higher expression levels in the older subjects, and training appears to attenuate expression of these genes. Not much is known about the function of CFAP61 except that it is highly expressed in skeletal muscle tissue and may be related to calcium signalling and/or energy conversion.^30^ C12orf75 may be related to cell proliferation and stem cell signalling.^31^

In our data frail subjects showed significantly higher expression levels of METTL21C compared to both the young and healthy older subjects, with a greater fold change difference between healthy and frail subjects than between the young and the older subjects (Table 3). Training decreases the mean expression of METTL21C in both groups, but this does not reach significance using our significance cut‐off. However, in the frail group it does reach a *q*‐value of 0.08 after training, showing a fold change of −1.58. METTL21C encodes for a protein–lysine methyltransferase belonging to a group of proteins that are involved in methylation of chaperone proteins, where METTL21C appears to methylate HSP70 and HSP90^32^ and has recently been found to be associated with skeletal muscle development.^33^ *In vitro* inhibition of METTL21C expression in myoblasts showed impaired myotube differentiation and calcium signalling, suggesting that METTL21C plays an important role in the function of muscle cells and possibly also overall quality of the muscle.

### Effect of prolonged resistance‐type exercise training

The majority of genes that significantly changed following prolonged resistance‐type exercise training showed a shift in the expression levels towards levels observed in the younger group (*Figure* 3). A previous study has shown a similar effect.^14^ Indeed, Melov *et al.* state that training reverses the effect of ageing. While there is a shift towards younger expression levels, this does not necessarily mean that there is reversal of ageing. A more likely explanation is that the skeletal muscle in these older subjects have been ‘detrained’ because of more sedentary lifestyle when compared with healthy younger controls. Physical inactivity is a major contributor to age‐related muscle loss and weakness and is one of the criteria of frailty.^2^ In this way participation in prolonged resistance‐type exercise training is likely to shift gene expression to younger levels. Furthermore, in our data the young subjects had higher muscle strength (Table 1). Training leads to subtle but consistent changes in the muscle transcriptome.^34^ Thus, a shift towards younger expression levels would be consistent with the increased strength after prolonged resistance‐type exercise training.

The genes that shift towards younger expression levels include genes related to the extracellular matrix, vascularization, glucose metabolism, and muscle contraction (Supporting Information, *Figures* S2, S5 and S6). The muscle biopsies were taken at least 3 days after the last training session. Thus, we are not observing acute effects of a single bout of resistance‐type exercise, but rather longer term consistent changes in gene expression. Notably absent among the changes induced by prolonged resistance‐type exercise training, however, are the primary differences observed when we compare young and older subjects: mitochondrial function. Possible explanations are that these changes are too subtle to pick up after 24 weeks of resistance‐type exercise training or that prolonged resistance‐type exercise training does not significantly affect these genes. Timing of the muscle biopsies relative to the last training session may also be a factor. It may be that expression of these mitochondrial genes only changes acutely after resistance‐type exercise rather than chronically.

Prolonged resistance‐type exercise training showed fewer significantly affected genes in the frail group. Part of this can be explained by the differences in treatment. The healthy older subjects had training sessions three times per week whereas the frail older subjects received two sessions per week. The load of the training was also lower for the frail subjects. However, it may also be that the frail older adults are less capable of adapting to the additional stress of prolonged resistance‐type exercise training. Fortunately, the frail subjects still showed a significant response to the training stimulus despite their less adaptive phenotype.^2^ Others have already reported that older adults in general have a decreased response to resistance‐type exercise on a transcriptome level,^35^ and this may also play a role in the smaller response in the frail older subjects compared with the healthy older subjects.

### Gene expression and muscle strength

Prolonged resistance‐type exercise training led to strength increases in all individuals to the point that training increased strength levels in the frail older subjects close to the levels observed in the healthy older subjects at baseline (*Figure* 1). However, it did not necessarily lead to increases in lean body mass in all individuals (*Figure* 2). This suggests that we primarily observed an increase in muscle quality, cross‐bridge cycling efficiency, calcium handling and/or neuromuscular adaptation rather than an increase in muscle cross‐sectional area. Our data provide evidence suggesting disturbances in axon guidance and muscle innervation in the older subjects.

We performed sPLS regression analysis to calculate leg strength based on expression levels of the genes that are robustly changed after prolonged exercise training in both groups in the baseline samples. Our rationale was that because these genes are changed in both groups after training, where the leg extension 1RM is significantly higher, that expression of these genes could also reflect muscle strength at baseline without training. We were able to build a reasonably accurate regression model to calculate leg extension 1RM at baseline based on gene expression (mean cross‐validation *R* ^2^ of 0.73 and RMSE of 17.7; *Figure* 4). Thus, expression of the genes robustly changed by exercise also seems to be indicative of muscle strength, not only after training but also prior to prolonged exercise training. This suggests that expression of these genes may be used as a biomarker to training status prior to study entry.

Several of the most important variables in our sPLS model for muscle strength belonged to the protocadherin gene cluster. Genes of the protocadherin gamma gene cluster were significantly different between frail older subjects, healthy older subjects and young subjects at baseline. Expression of these genes also went down after training in both groups. Older subjects had higher expression of this gene than young subjects, and expression was highest in the frail older subjects. There are good indications that this gene cluster is relevant for neuromuscular performance. Many of the genes from this cluster are also significantly changed after training in both groups. Protocadherin gamma genes ranked very highly in the variable importance for our correlative model for leg extension 1RM (Table 6).

Protocadherin gamma genes are primarily expressed in neural tissues such as the brain and the spinal cord and appears to be involved in axonal guidance.^36^ Protocadherin proteins show homeophilic binding to other protocadherin proteins, and in this way these proteins provide recognition sites for axonal binding. By expressing different protocadherin gamma genes from the gene cluster axons can be guided to different locations.^37^ In knockout mice these genes appear to be indirectly related to muscle function: knock‐out mice show severe muscle weakness and tremors, although this is attributed to loss of spinal motor neurons.^38^, ^39^ It may also be that it is expressed at the muscle side of the synapse to facilitate axon guidance towards muscle, and increased expression in this case is because of the denervation‐reinnervation cycles seen in ageing muscle. Therefore, we hypothesize that as muscle loses innervation it increases expression of the protocadherin gamma cluster to facilitate axon binding from other nerves.

Protocadherin gamma is not the only significant group of genes related to neuromuscular function that we found in our data. There are several other genes differentially expressed between frail and healthy older subjects that are related to the innervation of muscle, including acetylcholine esterase and kyphoscoliosis peptidase (KY). Both play important roles in the function of the neuromuscular junction.^40^, ^41^ The top differentially expressed gene at baseline, unc‐13 homolog C (UNC13C), is involved in neurotransmitter release.^42^, ^43^ Both MYLK4 and IGFN1 are also among the top significantly different genes between the three groups at baseline. Little is known about MYLK4 and IGFN1, but both have been indirectly associated with neuromuscular function. MYLK4 has been shown to be significantly downregulated in acetylcholine esterase knockout mice, together with KY, suggesting that it is somehow involved in the signal transduction.^44^ Like KY, IGFN1 has been associated with both muscle structure and neurological function.^45^, ^46^ This gene also binds EEF1A2, which is the gene associated with the wasted mouse phenotype.^46^, ^47^ This phenotype shows significant immunological and neuromuscular defects.^48^ IGFN1 has many splicing variants, which suggests that it plays a pleiotropic role in the muscle. Another indication of denervation is the increased expression of the perinatal myosin heavy chain isoform MYH8 in older subjects. Previous studies have found that expression of this gene is increased in tissue where the muscle fibres have lost innervation.^49^, ^51^

## Conclusions

Our data suggest a significant remaining plasticity of ageing skeletal muscle to adapt to regular resistance‐type exercise. Many age‐related changes in skeletal muscle gene expression are partially reversed by prolonged resistance‐type exercise training. Expression of the genes robustly changed following prolonged resistance‐type exercise training in frail and healthy older subjects did not only reflect the effect of training itself but also reflected muscle strength at baseline. Expression of the protocadherin gamma gene cluster is negatively correlated with muscle strength in our data and may be related to muscle denervation and re‐innervation.

### Clinical relevance

We have identified a gene cluster that may be related to denervation and re‐innervation cycles in the muscle. Loss of motor neurons has been suggested to play an important role in age‐related muscle weakness and sarcopenia but is unfortunately not yet fully understood. Prolonged resistance‐type exercise training was able to modulate the expression of protocadherin gamma. Hence, studying the expression of this gene may provide novel insights on whether or not denervation and re‐innervation is modulated by interventions or lifestyle factors such as nutrition and physical activity. Furthermore, in this paper we show that steady‐state gene expression analysis provides information on the strength of the muscle itself. This suggests that it could potentially be used as a tool to provide insight into muscle strength of a subject, but theoretically also into other muscle health‐related factors.

### Limitations

While we have a large sample size for such a study, we also have a very heterogeneous study population. The ratio of male to female among both groups of older adults is not entirely equal. We have adjusted for this in our statistical analyses where possible. Another limitation is that the muscle biopsies from the frail older adults were obtained from a study performed at Wageningen University, whereas the muscle biopsies from the healthy older adults and the young adults were obtained from studies performed at Maastricht University. Protocols for muscle biopsy collection and preparation in Wageningen are based on those from Maastricht and thus are very similar, but there may still be some bias that we cannot account for statistically. The microarray analyses were performed within the same laboratory at the same time by the same technician, which means that batch effects should be minimal. The protocols for the prolonged resistance‐type exercise training were slightly different for the frail older adults and the healthy older adults. The primary difference being that the healthy older adults trained three times per week and the frail older adults trained twice per week. As a consequence, the training stimulus for the frail older adults was somewhat lower in these individuals, and this could partially explain the decreased response among the frail older subjects. This difference in treatment also prevented us to compare the training responses in both groups directly.

## Conflict of interest

J.A.. Timmons is a Founding Director of XR Genomics, a Personalised Health and Fitness company. John van Duynhoven is employed by a company that manufactures and markets food products. The other authors have no competing interests to declare.

## Supporting information

Supporting info item

Supporting info item

Supporting info item

Supporting info item

Supporting info item

Supporting info item

Supporting info item

Supporting info item

Supporting info item

Supporting info item
