# Supplementary figures and images for: Expression of protocadherin gamma in skeletal muscle tissue is associated with age and muscle weakness
Source: J Cachexia Sarcopenia Muscle. 2016 Feb 2;7(5):604–14. doi: 10.1002/jcsm.12099 (PMC4863830; doi:10.1002/jcsm.12099)

FE - training

FE vs. HE

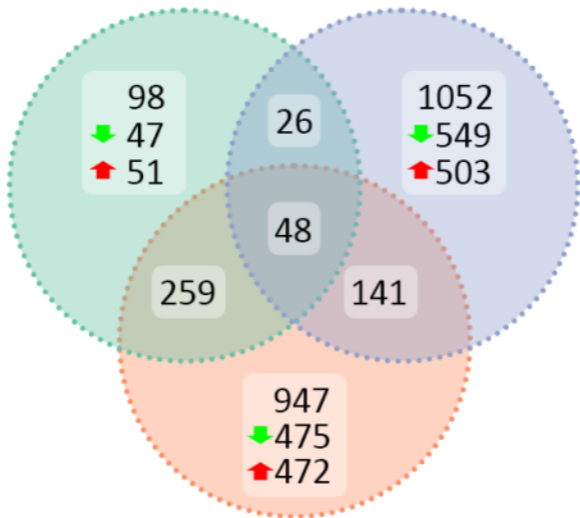

HE - Training

Supplement: Supplementary file 1 — Supporting info item [file JCSM-7-604-s001.pdf]

FE vs. YO

HE vs. YO

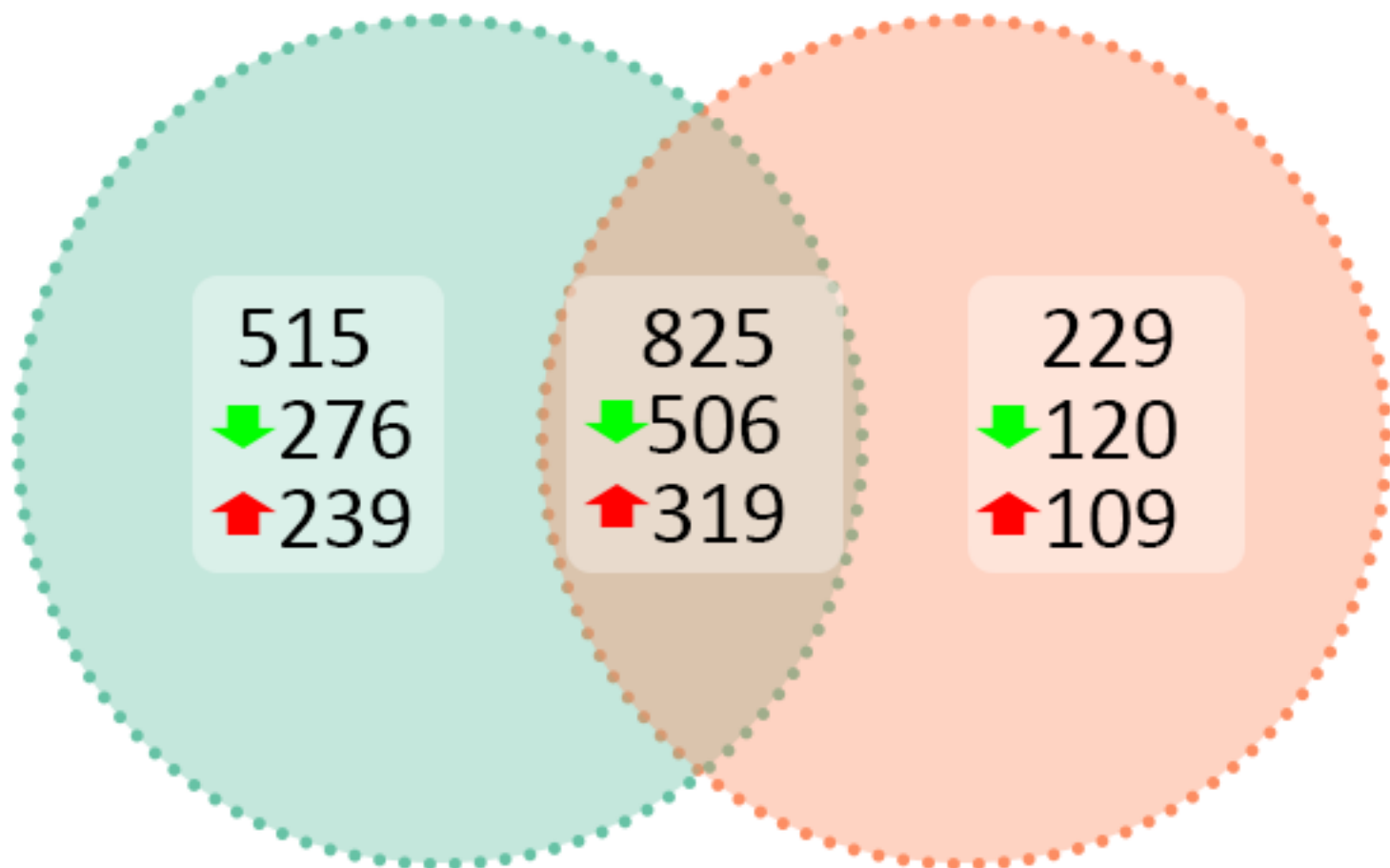

Supplement: Supplementary file 2 — Supporting info item [file JCSM-7-604-s002.pdf]

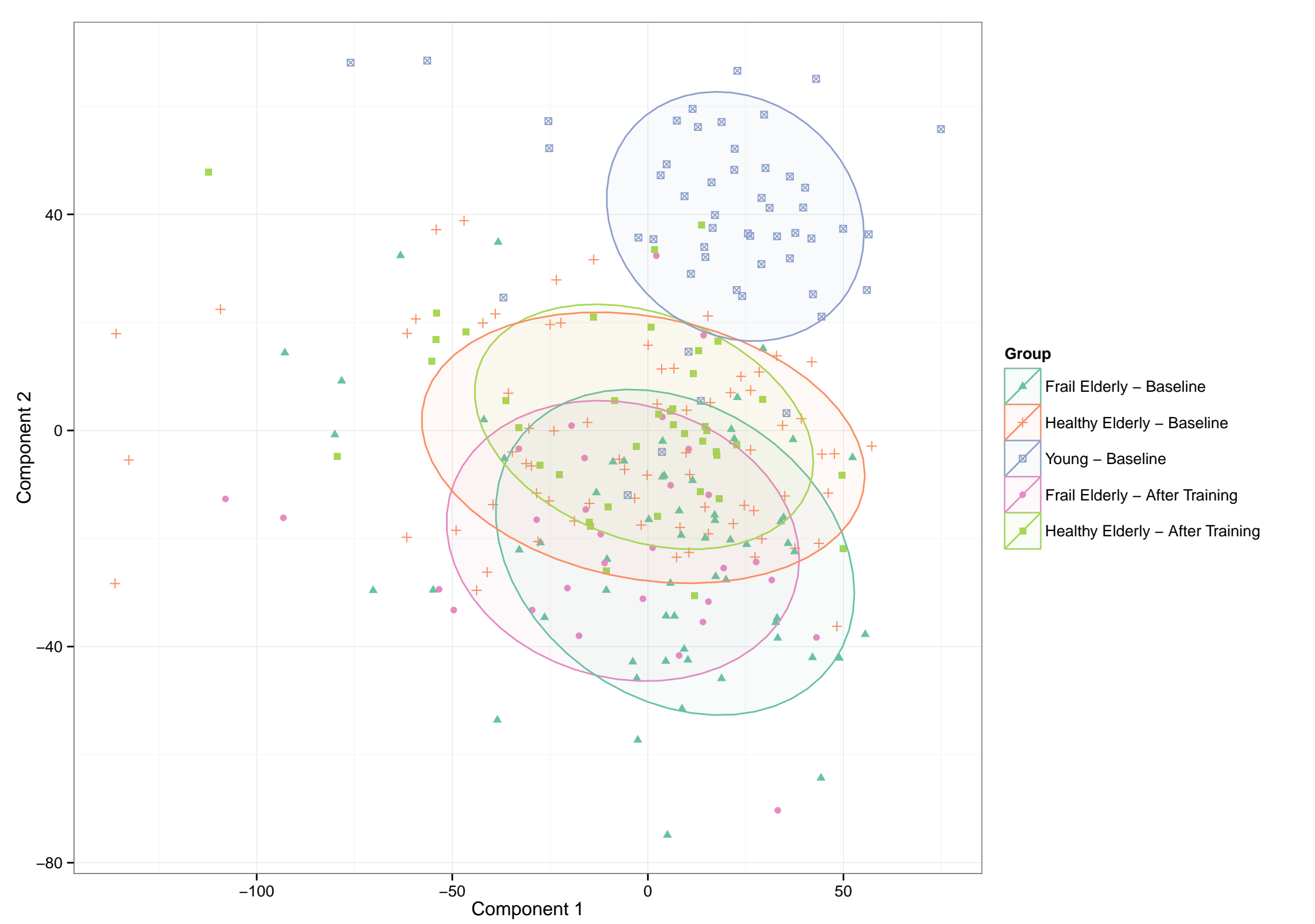

Supplement: Supplementary file 4 — Supporting info item [file JCSM-7-604-s004.pdf]

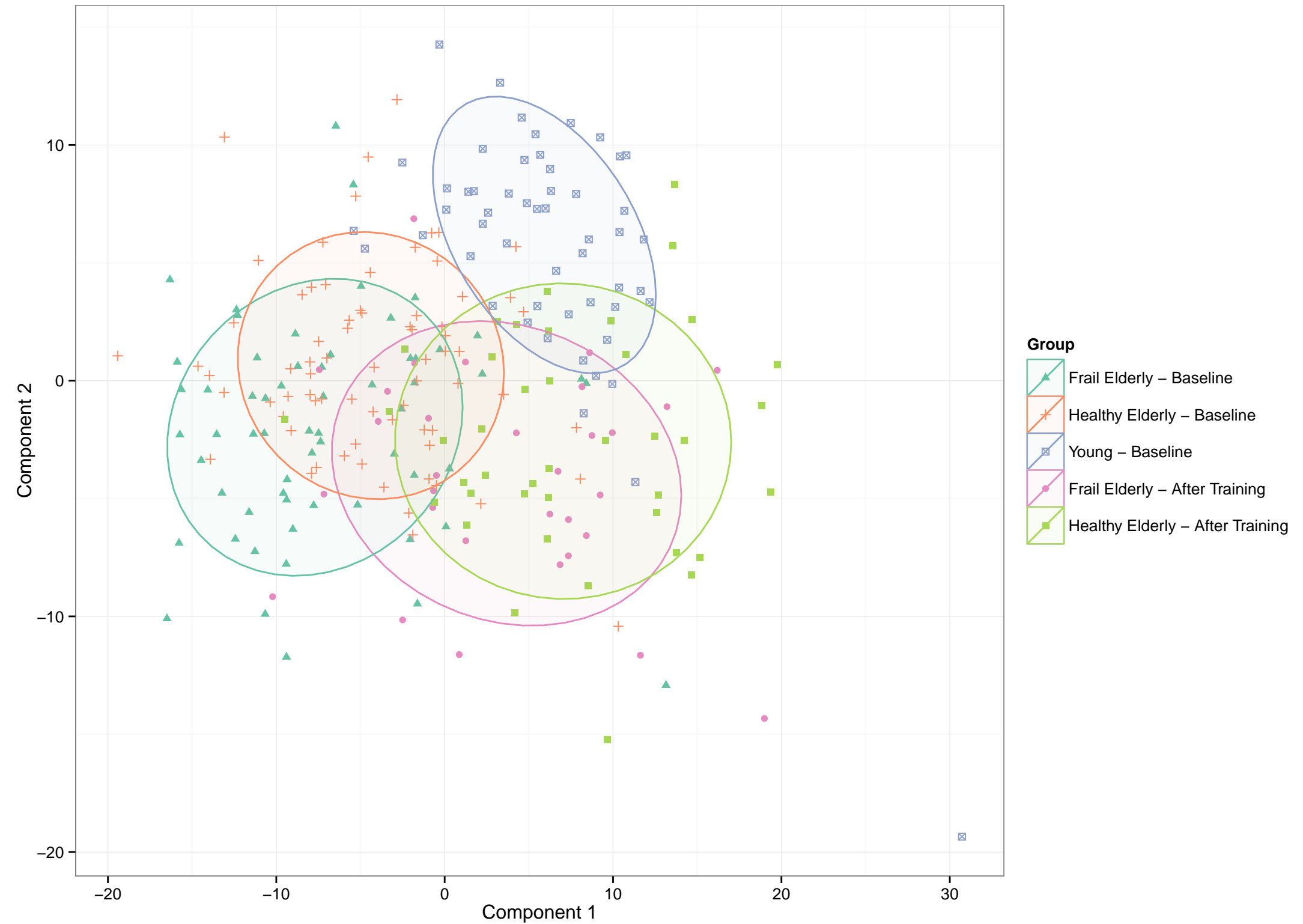

Supplement: Supplementary file 5 — Supporting info item [file JCSM-7-604-s005.pdf]

Negative correlation between PCDHG gene expression and leg extension 1RM

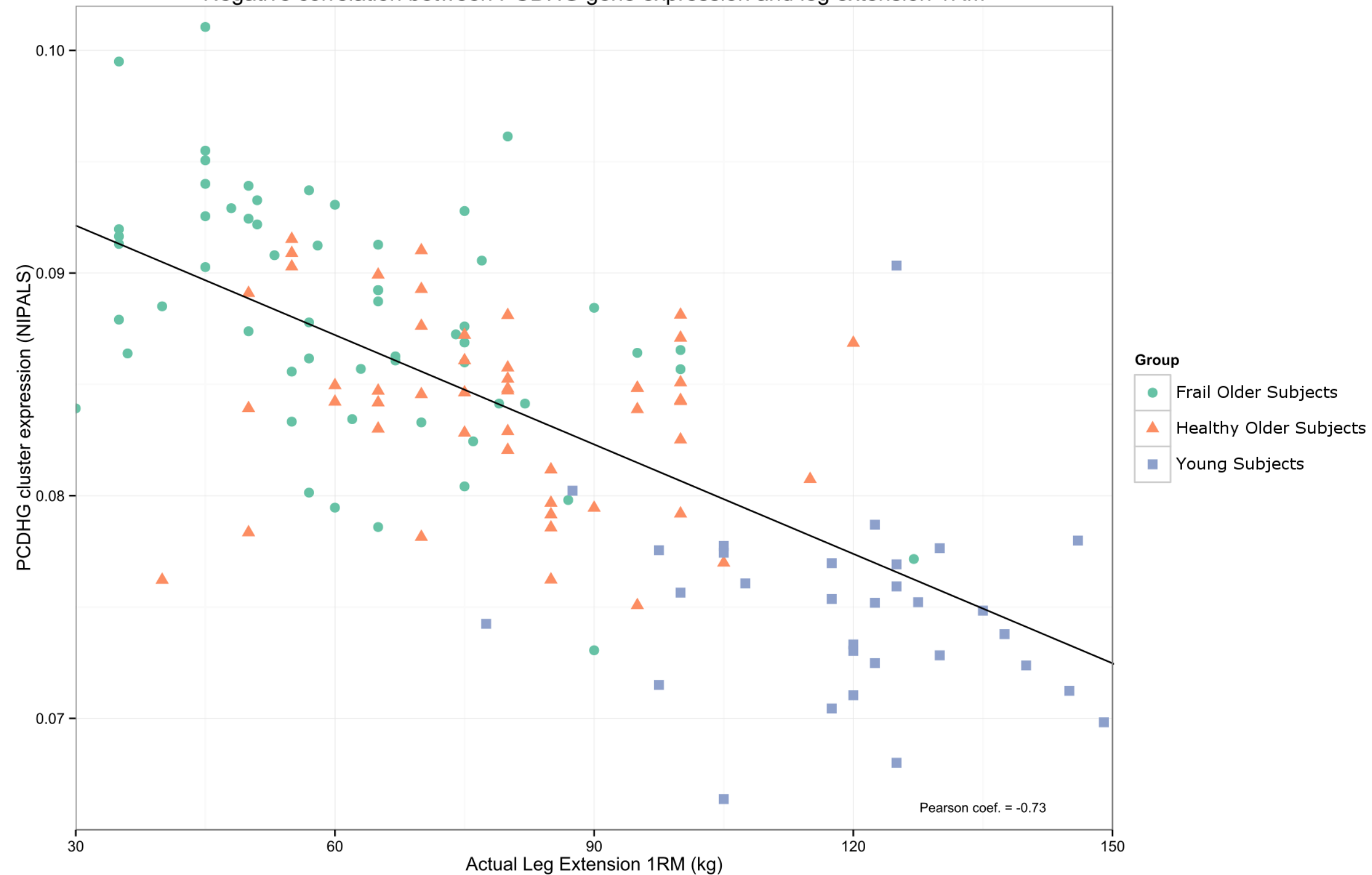

Supplement: Supplementary file 8 — Supporting info item [file JCSM-7-604-s008.pdf]

Negative 10 log of p-value

0 1 2 3 4 5 6 7 8 9 10 11 12

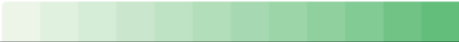

Supplement: Supplementary file 9 — Supporting info item [file JCSM-7-604-s009.pdf]

Activation z-score

-2      -1.5      -1      -0.5      0      0.5      1      1.5      2

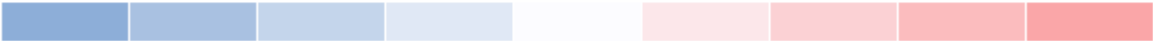

Supplement: Supplementary file 10 — Supporting info item [file JCSM-7-604-s010.pdf]
