# Supplementary material for: Expression of protocadherin gamma in skeletal muscle tissue is associated with age and muscle weakness
Source: J Cachexia Sarcopenia Muscle. 2016 Feb 2;7(5):604–14. doi: 10.1002/jcsm.12099 (PMC4863830; doi:10.1002/jcsm.12099)
Supplement: Supplementary file 3 — Supporting info item [file JCSM-7-604-s003.pdf]

Canonical Pathway	FE training	HE training	FE vs HE	FE vs YO	HE vs YO
Oxidative Phosphorylation					
Mitochondrial Dysfunction					
Hepatic Fibrosis / Hepatic Stellate Cell Activation					
Glycolysis I					
Granulocyte Adhesion and Diapedesis					
Agranulocyte Adhesion and Diapedesis					
G Protein Signaling Mediated by Tubby					
Gluconeogenesis I					
TCA Cycle II (Eukaryotic)					
Intrinsic Prothrombin Activation Pathway					
Role of Osteoblasts, Osteoclasts and Chondrocytes in Rheumatoid Arthritis					
CCR5 Signaling in Macrophages					
Leukocyte Extravasation Signaling					
Sphingosine-1-phosphate Signaling					
Aspartate Degradation II					
Glutamate Receptor Signaling					
Tec Kinase Signaling					
Inhibition of Matrix Metalloproteases					
Human Embryonic Stem Cell Pluripotency					
Allograft Rejection Signaling					
Aldosterone Signaling in Epithelial Cells					
Calcium Signaling					
OX40 Signaling Pathway					
Type I Diabetes Mellitus Signaling					
Acetyl-CoA Biosynthesis I (Pyruvate Dehydrogenase Complex)					
Gustation Pathway					
Regulation of the Epithelial-Mesenchymal Transition Pathway					
Glutamate Degradation II					
L-cysteine Degradation I					
Aspartate Biosynthesis					
Oxidative Ethanol Degradation III					
Communication between Innate and Adaptive Immune Cells					
Axonal Guidance Signaling					
Tryptophan Degradation X (Mammalian, via Tryptamine)					
Superpathway of Melatonin Degradation					
Dopamine Degradation					
Thioredoxin Pathway					
Melatonin Degradation II					
IL-8 Signaling					
Ethanol Degradation IV					
