# Supplementary material for: Expression of protocadherin gamma in skeletal muscle tissue is associated with age and muscle weakness
Source: J Cachexia Sarcopenia Muscle. 2016 Feb 2;7(5):604–14. doi: 10.1002/jcsm.12099 (PMC4863830; doi:10.1002/jcsm.12099)
Supplement: Supplementary file 6 — Supporting info item [file JCSM-7-604-s006.pdf]

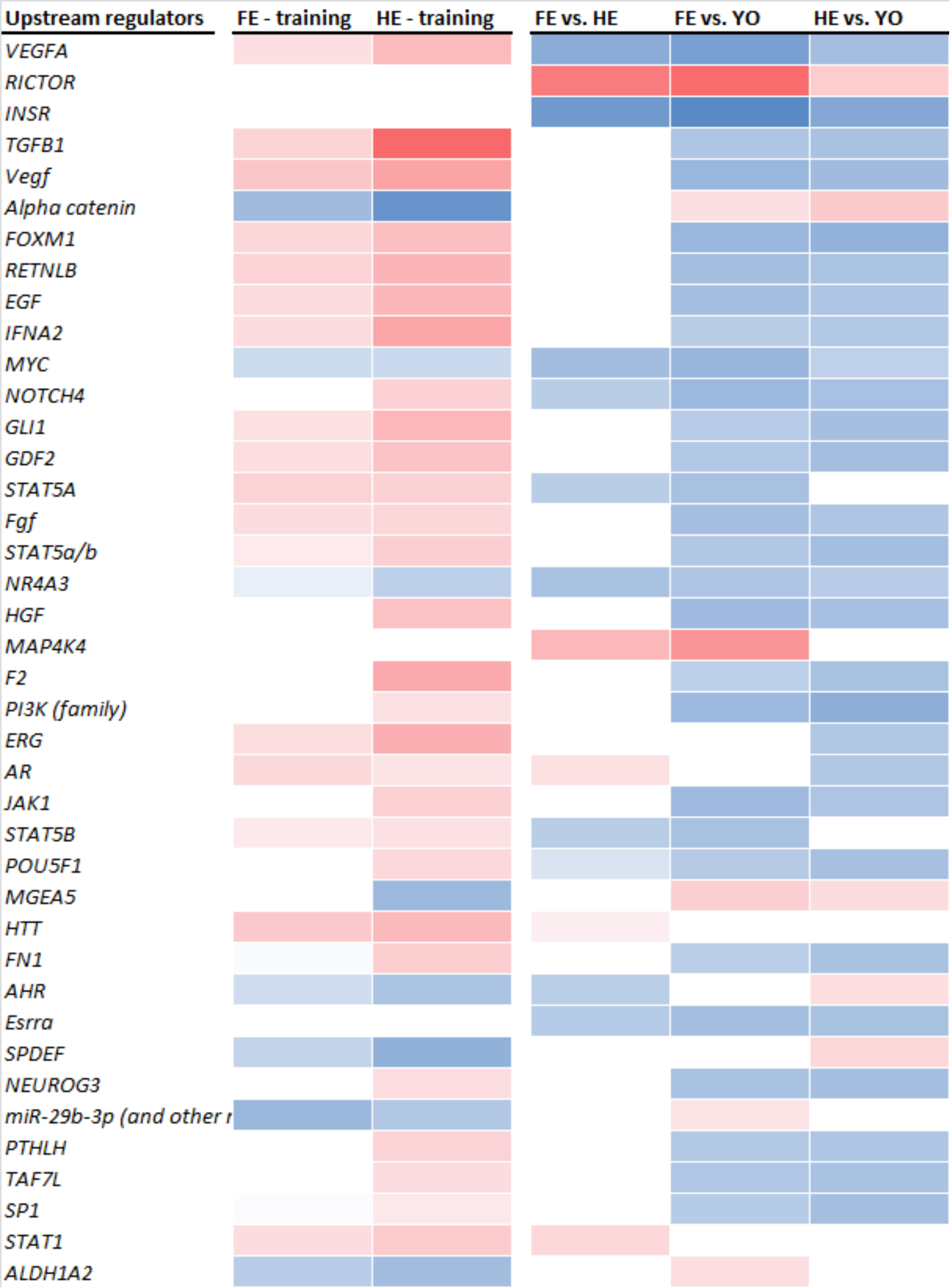
Upstream regulators
FE - training
HE - training
FE vs. HE
FE vs. YO
HE vs. YO
VEGFA
RICTOR
INSR
TGFB1
Vegf
Alpha catenin
FOXM1
RETNLB
EGF
IFNA2
MYC
NOTCH4
GLI1
GDF2
STAT5A
Fgf
STAT5a/b
NR4A3
HGF
MAP4K4
F2
PI3K (family)
ERG
AR
JAK1
STAT5B
POU5F1
MGEA5
HTT
FN1
AHR
Esrra
SPDEF
NEUROG3
miR-29b-3p (and other r
PTHLH
TAF7L
SP1
STAT1
ALDH1A2
