# Supplementary material for: Expression of protocadherin gamma in skeletal muscle tissue is associated with age and muscle weakness
Source: J Cachexia Sarcopenia Muscle. 2016 Feb 2;7(5):604–14. doi: 10.1002/jcsm.12099 (PMC4863830; doi:10.1002/jcsm.12099)
Supplement: Supplementary file 7 — Supporting info item [file JCSM-7-604-s007.pdf]

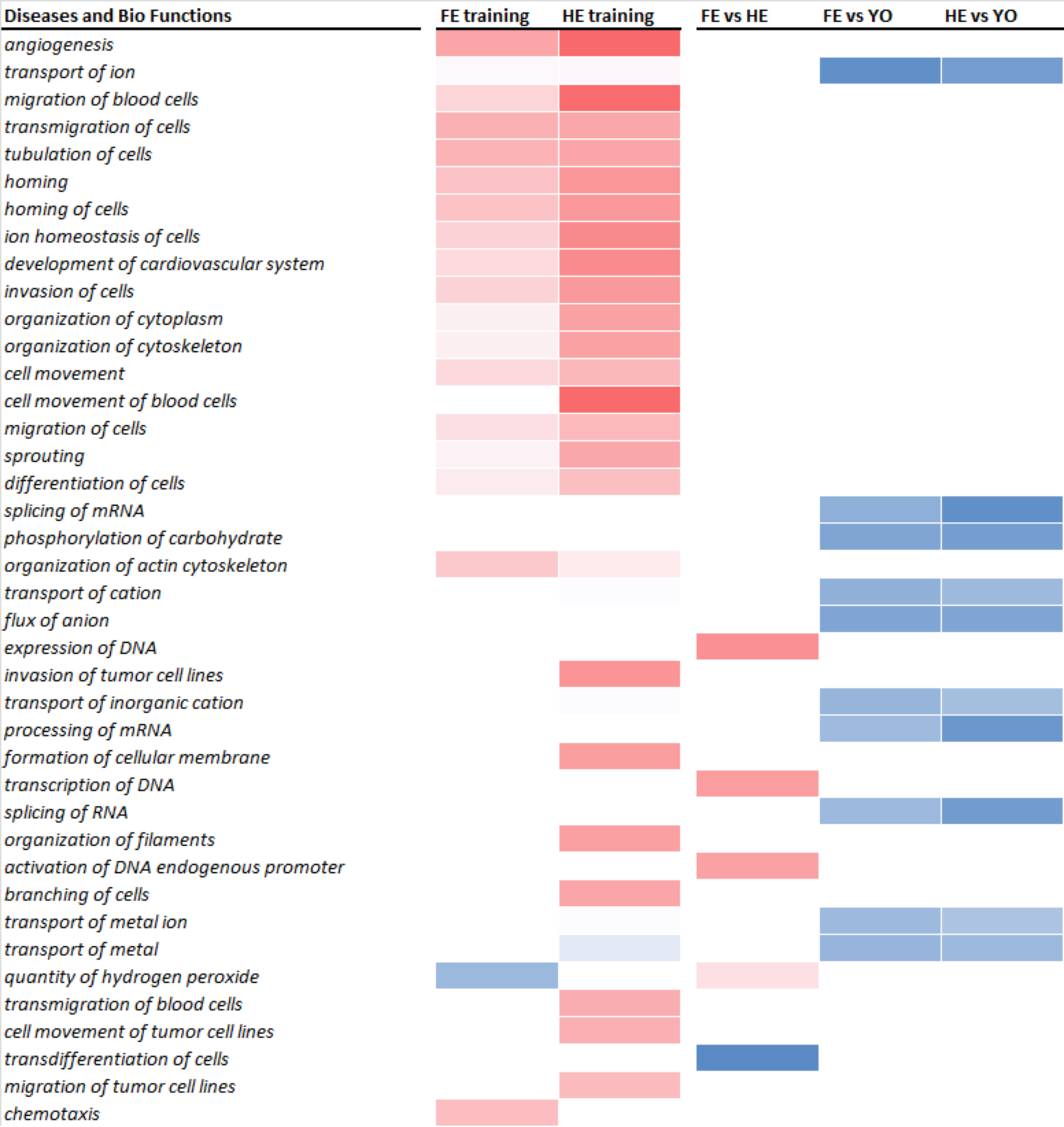
Diseases and Bio Functions
FE training
HE training
FE vs HE
FE vs YO
HE vs YO
angiogenesis
transport of ion
migration of blood cells
transmigration of cells
tubulation of cells
homing
homing of cells
ion homeostasis of cells
development of cardiovascular system
invasion of cells
organization of cytoplasm
organization of cytoskeleton
cell movement
cell movement of blood cells
migration of cells
sprouting
differentiation of cells
splicing of mRNA
phosphorylation of carbohydrate
organization of actin cytoskeleton
transport of cation
flux of anion
expression of DNA
invasion of tumor cell lines
transport of inorganic cation
processing of mRNA
formation of cellular membrane
transcription of DNA
splicing of RNA
organization of filaments
activation of DNA endogenous promoter
branching of cells
transport of metal ion
transport of metal
quantity of hydrogen peroxide
transmigration of blood cells
cell movement of tumor cell lines
transdifferentiation of cells
migration of tumor cell lines
chemotaxis
